# A structural equation model-based study on the status and influencing factors of acute exacerbation readmission of elderly patients with chronic obstructive pulmonary disease within 30 days

**DOI:** 10.1186/s12890-022-02093-w

**Published:** 2022-08-04

**Authors:** Hong-Yan Lu, Rui Zhang, Yan Chang, Xiao-Na Zhang, Jie Zhao, Xin-Dan Li, Xiang-Kai Feng

**Affiliations:** 1 Department of Nursing, The General Hospital of Ningxia Medical University, No. 804 Shengli Street, Xingqing District, Yinchuan, 750004 China; 2 Department of Nursing, XiangYang First People’s Hospital, XiangYang, 441002 China

**Keywords:** Chronic obstructive pulmonary disease, Acute exacerbation readmission within 30 days, Influencing factors, Structural equation model

## Abstract

**Objective:**

To investigate the circumstances that lead to acute exacerbation readmission of elderly patients with chronic obstructive pulmonary disease (COPD) within 30 days and to explore the influencing factors of readmission using a structural equation model to provide evidence for medical staff so that effective intervention measures can be taken.

**Methods:**

The convenience sampling method was used to select 1120 elderly patients with COPD from the respiratory departments of thirteen general hospitals in the Ningxia region, China, from April 2019 to August 2020, who then completed a survey questionnaire. The survey questionnaire contained a general data questionnaire and the modified Medical Research Council, activities of daily living, geriatric depression scale and COPD assessment test scales.

**Results:**

The readmission rate of patients with COPD presenting with acute exacerbation within 30 days was determined to be 21.52%. Therefore, the modified model measures data accurately. The results showed that seasonal factors, family rehabilitation, age factors and overall health status were direct factors in the acute exacerbation readmission of patients with COPD within 30 days of hospital discharge. Smoking is not only a direct factor for acute exacerbation readmission within 30 days but also an indirect factor through disease status; disease status and chronic disease are not only direct factors for acute exacerbation readmission within 30 days but also indirect factors through the patient’s overall health status.

**Conclusions:**

The rate of patients with COPD presenting with acute exacerbation within 30 days is high; while taking measures to prevent readmission based on influencing factors that directly impact admission rates, attention should also be paid to the interaction between these factors.

## Introduction

According to the Global Initiative on Chronic Obstructive Pulmonary Disease (2019), chronic obstructive pulmonary disease (COPD) is a disease with one of the highest morbidity and mortality rates worldwide and has become a major challenge to public health management [1], and acute exacerbation of COPD is an event that adversely affects disease management [2]. Studies have shown that the readmission rate of patients with acute exacerbation of COPD within 30 days is 6.70–7.54%, among which the readmission rate of elderly patients is 18.4%, with age being an independent influencing factor [3–5]. Due to the short acute exacerbation readmission cycle of COPD of 30 days, readmission not only seriously damages lung function and increases the risk of death but also requires a large number of medical resources, which is one of the evaluation indicators of hospital medical quality in the United States [6]. Studies have shown that managing the influencing factors of readmission can lead to its avoidance in some populations [7]. In the past, regression analysis was used to discuss influencing factors. However, indicators such as disease, smoking and health statuses, as well as family rehabilitation, could not be measured directly. When regression analyses were used in the past, these indicators were usually integrated into just one, which led to certain information being lost. At the same time, the regression analysis method considers whether independent variables directly affect the dependent variable as the estimation target without considering the internal interaction between independent variables [8]. The structural equation model can compensate for this deficiency in the traditional regression analysis method and provides several benefits over univariate and multiple logistic regression methods. It is possible to calculate many independent and dependent variables at once, and measurement mistakes may be decreased by processing certain possible variables that cannot be directly measured. It is also possible to investigate the pathophysiology of illnesses and assess the routes and indirect influences of many components [8]. The structural equation model is thus increasingly used when examining the intricate interactions between risk factors linked to the development of clinical outcomes. Therefore, this study investigates the status of acute exacerbation readmission of elderly Chinese elderly patients with COPD within 30 days and analyses the influencing factors in readmission using the structural equation model.

## Methods

### Study population

Patients with COPD who met the inclusion and exclusion criteria were selected from the respiratory departments of thirteen general hospitals in the Ningxia region, China, from April 2019 to August 2020. Exacerbation was defined as an acute worsening of the respiratory symptoms of COPD that exceeded common daily variations and led to an emergency room visit or hospitalisation (GOLD, 2022). Inclusion criteria: (1) patients who met clinical COPD diagnostic criteria (GOLD, 2022) and had been diagnosed; (2) age ≥ 60; (3) patients who gave informed consent and voluntarily participated in the study. Exclusion criteria: (1) patients with severe audio-visual impairment and an inability to communicate; (2) patients in poor condition who had difficulties completing the survey questionnaire.

According to the sample size calculation formula of the cross-sectional study: $$n = \frac{{Z^{2} {\raise0.7ex\hbox{$\alpha $} \!\mathord{\left/ {\vphantom {\alpha 2}}\right.\kern-\nulldelimiterspace} \!\lower0.7ex\hbox{$2$}}\pi \left( {1 - \pi } \right)}}{{\delta^{2} }}$$ and according to previous studies [5], *π* = 0.184. *α* = 0.05, Z_0.05/2_ = 1.96, *δ* = 0.025 and n = 923. One thousand two hundred sixty-eight cases were included in the study; 86 incomplete questionnaires were excluded, as well as 62 patients who were lost to the study, with 1120 cases finally obtained as the study sample. The ratio of sample size to index was 1120:21, which met the requirement that the sample size of the structural equation model is ten times larger than the measurement variable (210), and the structural equation model could therefore be used [7]. Elderly patients with COPD were classified as non-readmitted or readmitted based on whether they were readmitted for acute exacerbation within 30 days of discharge.

### Data collection and measurement

The relevant factors affecting acute exacerbation readmission of elderly patients with COPD within 30 days were collected, including disease, smoking and overall health statuses, as well as information relating to family rehabilitation. Based on these data, the questionnaire and survey contents were designed.

The researcher designed the general data variables according to the purpose and content of the study through a literature review and preliminary survey, including age, gender, and smoking status (including duration and quantity); information regarding whether the patient suffered from diabetes, hypoalbuminemia, high blood pressure or coronary heart disease (CHD); further information consisting of respiratory frequency, SaO2 PO2 and PCO2 rates, details of any at-home oxygen therapy, rehabilitation exercise routine or regular medication taken; the number of exacerbations in hospital and readmissions in the past year; and the readmission season. The modified Medical Research Council (mMRC) scale was used to determine the severity of dyspnoea in the survey participants. The scale was divided into five levels. Level 0: difficulty breathing only during strenuous activity; level 1: shortness of breath when walking briskly on flat ground or walking uphill; level 2: needing to stop to rest due to shortness of breath; level 3: needing to stop to rest after a few minutes of walking or after walking on flat ground for about 100 m; level 4: unable to leave the house because of severe breathing difficulties or difficulty breathing when putting on or taking off clothes. Patients with mMRC breathlessness scores ≥ 2 were defined as having chronic breathlessness [9]. Activities of daily living (ADL) scores were used to evaluate patients’ ability to complete daily living activities. The maximum score was 100 points with an evaluation standard of 81–100 points (completely self-reliant), 61–80 points (mild dysfunction with the ability to complete daily activities independently), 41–60 points (moderate dysfunction with assistance needed to complete general tasks) and ≤ 40 points (severely dysfunctional or wholly dependent and most daily activities could not be completed or required assistance) [10]. The geriatric depression scale (GDS) was used to measure depression levels over the last week. The maximum score was 30, and the assessment standard was 0–10 (normal or no depression), 11–20 (likely to have depressive symptoms) and 21–30 (depression) [11]. The mini nutritional assessment-short form was used to evaluate the dietary status of patients using eight questions, with a maximum score of 14: 12–14 (normal nutritional status), 8–11 (risk of malnutrition) and 0–7 (undernourished) [12]. The COPD assessment test (CAT) scale was used to assess the severity of COPD and included eight questions with a maximum score of 40 points. The total scores were classified as less than 10 (mild illness), 10–20 (moderate illness), 20–30 (serious illness) and more than 30 (severe illness) [9].

### Ethical statement

This study was approved by the ethics review institution of the General Hospital of Ningxia Medical University (2020-643), complying with the declaration of Helsinki. Prior to data collection, consent and cooperation agreements were obtained from nine hospital administrators and departments, and prior to participation, all patients taking part in the study signed written informed consent forms.

### Data collection

The patients who could cooperate with the study were surveyed by questionnaire and followed up 30 days after discharge. The follow-up information included whether the patient had been readmitted due to acute exacerbation, whether the patient had taken any regular medication and whether the patient had completed any rehabilitation exercises. The readmission season was also recorded.

### Quality control

The researchers completed a preliminary survey of forty-eight elderly patients with COPD. Investigators underwent training to ensure data consistency, and the training included research objectives, questionnaire completion requirements and the evaluation methods of each scale. The trained investigators made a point-by-point statement and completed a questionnaire based on the patient’s answers. On-site release and recovery, check whether there is any leakage, check, and recall. The data were recorded by two researchers, and questionnaires with errors or omissions exceeding 20% or of complete similarity were excluded.

### Data analysis

Epidata 3.1 was used for data entry, and SPSS25.0 was used for data processing and exploratory factor analysis. The frequency and composition ratio was used for the statistical description. AMOS25.0 software was used to tailor, evaluate and modify the model, establish the final model and explore the path coefficient among variables. Bartlett’s sphericity test was used to examine the suitability of the study data for exploratory factor analysis. Input variables included the length, number of smoking, smoking status, gender, diabetes, hypoalbuminemia, hypertension, CHD, mMRC, CAT, ADL, depression status, nutritional status, respiratory frequency, SaO2, PO2 and PCO2 rates, home oxygen therapy, rehabilitation exercise, regular medication, age, and the number of exacerbations, as well as the readmission season, in the past year. After in-depth consideration of the correction index, critical ratio, path coefficient and standardised residual, the hypothesised model was tested for goodness of fit and maximum likelihood estimation and was modified according to the correction index and the actual study environment; a model that met the adaptation requirements was thereby obtained. When the coefficient was higher than zero, the factor had a positive regulatory effect. Otherwise, it had a negative regulatory effect. *P* < 0.05 was considered to be of statistical significance.

## Results

### General information regarding readmission for acute exacerbation of COPD within 30 days

In this study, the data received from a total of 1120 elderly patients with COPD were investigated, and the study concluded that 241 patients (21.52%) were readmitted due to acute exacerbation of COPD within 30 days. Eight hundred seventy-nine cases (78.48%) were not readmitted. The basic situation is shown in Table 1.

**Table 1:** Basic situation of elderly COPD patients (n = 1120)

Items		n	Proportion (%)
Sex	Male	683	60.98
	Female	437	39.02
Age (years)	60–69	404	36.07
	70–79	453	40.45
	≥ 80	263	23.48
Hypertension	No	617	55.09
	Yes	503	44.91
Diabetes	No	885	79.02
	Yes	235	20.98
Coronary heart disease(CHD)	No	860	76.79
	Yes	260	23.21
Hypoproteinemia	No	1097	97.95
	Yes	23	2.05
Hospitalized with acute	< 2	618	55.18
Exacerbation of COPD in	≥ 2	502	44.82
*Past 1 year (times)*			
Smoking status	Never smoking	641	57.23
	To give up smoking	395	35.27
	Smoking	84	7.50
Smoking quantity (years)	< 10	691	61.70
	10–20	165	14.73
	> 20	264	23.57
Smoking duration (cigarettes/day)	0	642	57.32
	1–10	217	19.38
	11–20	174	15.54
	≥ 21	87	7.76
PaCO2 (mmHg)	< 35	347	30.98
	35–45	499	44.55
	> 45	274	24.47
PaO2 (mmHg)	< 80	867	77.41
	80–100	253	22.59
SaO2 (%)	< 95	876	78.21
	≥ 95	244	21.79
Respiration rate (times/minute)	< 16	111	9.91
	16–20	483	43.13
	> 20	526	46.96
Seasonal factors	Spring	231	20.63
	Summer	241	21.52
	Autumn	299	26.70
	Winter	349	31.16
Long-term home oxygen therapy	No	806	71.96
	Yes	314	28.04
Regular medication	No	860	76.79
	Yes	260	23.21
Rehabilitation exercise	No	774	69.11
	Yes	346	30.89
CAT	Slight symptoms	40	3.57
	Medium	380	33.93
	Serious	586	52.32
	Very serious	114	10.18
mMRC	Level 0	113	10.09
	Level 1	259	23.12
	Level 2	368	32.86
	Level 3	285	25.45
	Level 4	95	8.48
ADL	Completely independent	167	14.91
	Mild dysfunction	650	58.04
	Moderate dysfunction	196	17.50
	Severe dysfunction or complete dependence	107	9.55
GDS	There is no depression	481	42.95
	Mild depression	479	42.77
	Moderate to severe depression	160	14.28
Nutritional condition	Normal nutritional status	307	27.41
	Risk of malnutrition	502	44.82
	Malnutrition	311	27.77

### Construction of the initial structural equation model

#### Construction of a measurement model using exploratory factor analysis

The measurement model was composed of latent and observed variables. Common factors were extracted through exploratory factor analysis to find the observed variables that corresponded to the latent variables affecting acute exacerbation readmission of elderly patients with COPD within 30 days. The variables in Table 1 were included in the exploratory factor analysis. The goodness-of-fit tests of the hypothesised model showed a Kaiser–Meyer–Olkin (KMO) score of KMO = 0.768 > 0.6 and the approximate *χ2* = 4366.074 (*df* = 253, *P* < 0.001) of Bartlett’s sphericity test, indicating that there was a correlation between variables in this study, meeting the condition of exploratory factor analysis. According to the Eigenvalues > 1 and combined with lithotripsy, the common factor number extracted was seven, and the total variation of accumulative interpretation data was 61.11%. The factor load matrix after rotation is shown in Table 2.

**Table 2:** Matrix after rotation of factor load

Characteristics	Common factor 1	Common factor 2	Common factor 3	Common factor 4	Common factor 5	Common factor 6	Common factor7
Smoking duration	0.910	–	–	–	–	–	–
Smoking quantity	0.900	–	–	–	–	–	–
Smoking status	0.877	–	–	–	–	–	–
Sex	0.708	–	–	–	–	–	–
Diabetes	–	0.841	–	–	–	–	–
Hypoproteinemia	–	0.808	–	–	–	–	–
Hypertension	–	0.779	–	–	–	–	–
CHD	–	0.734	–	–	–	–	–
mMRC	–	–	0.758	–	–	–	–
CAT	–	–	0.740	–	–	–	–
ADL	–	–	0.710	–	–	–	–
GDS	–	–	0.606	–	–	–	–
Nutritional condition condition	–	–	0.563	–	–	–	–
Respiration rate	–	–	–	0.777	–	–	–
SaO2	–	–	–	0.773	–	–	–
PO2	–	–	–	0.685	–	–	–
PCO2	–	–	–	0.577	–	–	–
Home oxygen therapy	–	–	–	–	0.772	–	–
Rehabilitation exercise	–	–	–	–	0.730	–	–
Regular medication	–	–	–	–	0.719	–	–
Number of	–	–	–	–	–	0.783	–
Hospitalizations							
In past 1 year							
Age factors	–	–	–	–	–	0.558	–
Seasonal factors	–	–	–	–	–	–	0.957

The first factor load included smoking status (including duration and quantity) and gender, and this factor was used as the observation variable to construct a latent variable named smoking status. The second factor load included diabetes, hypoproteinaemia, hypertension and CHD, and this factor was used as the observation variable to construct a latent variable named chronic disease. The third factor load included mMRC, CAT, ADL, GDS scores and nutritional condition, and this factor was used as the observation variable to construct a latent variable named overall status. The fourth factor load included respiration rate and SaO2, PO2 and PCO2 intake, and this factor was used as the observation variable to construct a latent variable named disease status. The fifth factor load included in-home oxygen therapy, rehabilitation exercise and regular medication, and this factor was used as the observation variable to construct a latent variable named family rehabilitation. The sixth factor load included age and number of hospitalisations, with the number of acute exacerbations of COPD in the past year including a larger load of this variable within the same factor, which may be due to the strong correlation between age and acute exacerbation. Therefore, the two factors were set to reflect the potential variable of age factors. Finally, the seventh factor load included seasonal influencing factors.

#### The initial model

**Fig. 1:**
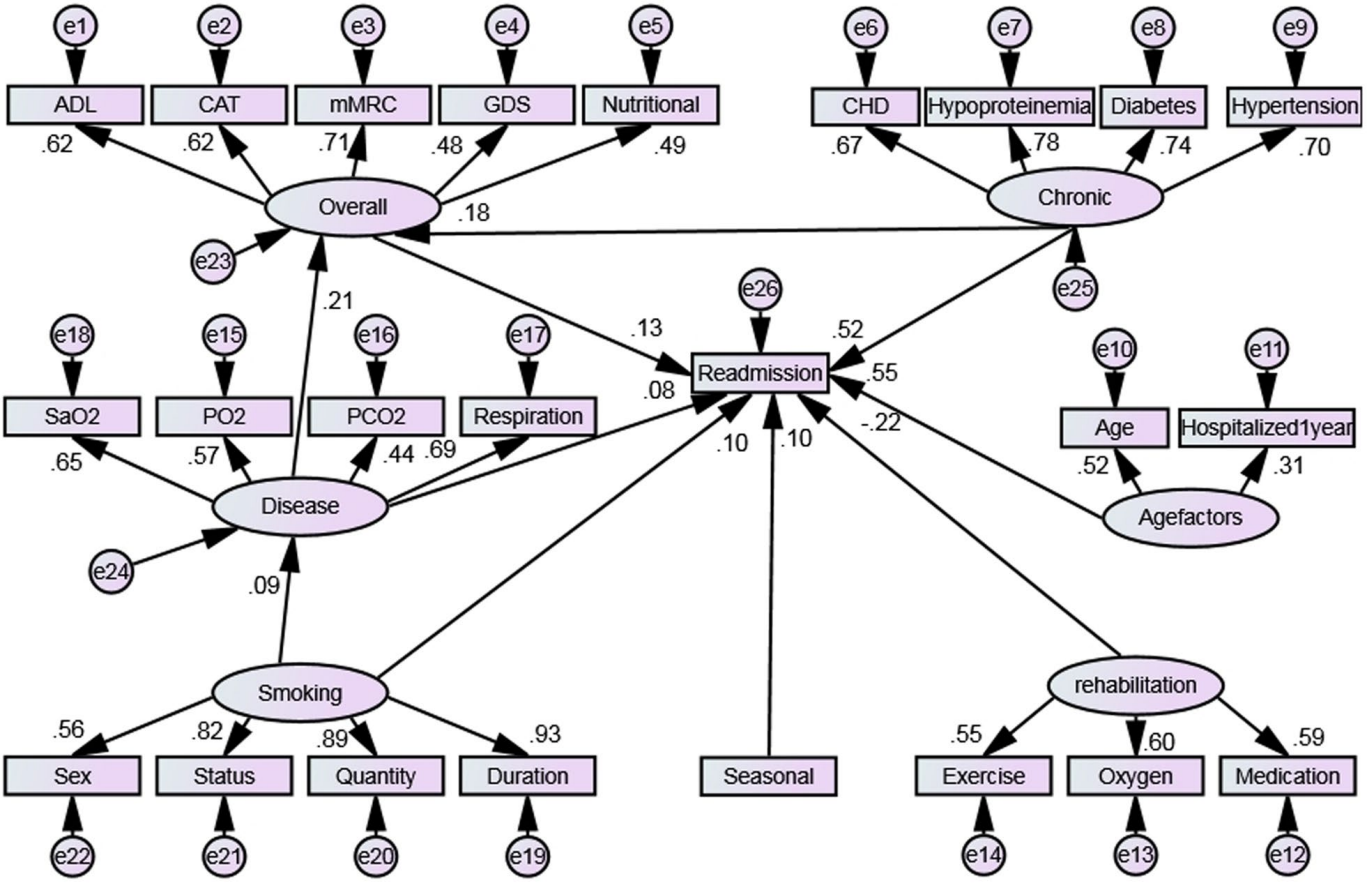
Structural equation model of influencing factors of acute exacerbation readmission in elderly COPD patients within 30 days (initial)

Based on the completed exploratory factor analysis, combined with literature and professional knowledge, the initial structural equation model was constructed, and the standardised path diagram is shown in Fig. 1.

### Evaluation and modification of the model

**Fig. 2:**
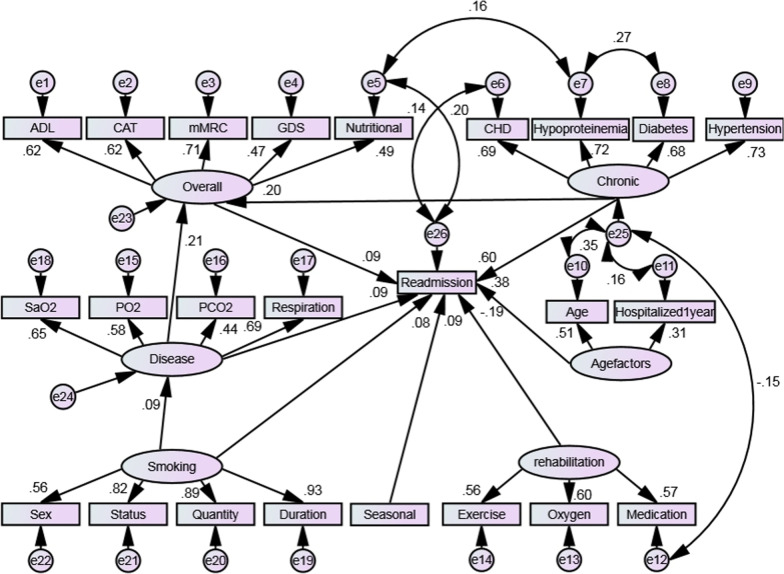
Structural equation model of influencing factors of acute exacerbation readmission in elderly COPD patients within 30 days (Modified)

It was found that the model was recognisable according to the established initial model path diagram. However, the relevant index was not ideal and failed to meet the adaptation standard, as shown in Table 3. After in-depth consideration of the correction index, critical ratio, path coefficient and the standardised residual, the model was modified according to the correction index and actual study conditions. A model that met the adaptation requirements was thereby obtained, as shown in Table 3. The normalised path diagram is shown in Fig. 2. The results of path parameters showed that each path was consistent with 30-day readmission (*P* < 0.05).

**Table 3:** Fitting index standards and results of structural equation models

Project	χ2/df	RMSEA	NFI	RFI	TLI	CFI	PRATIO	PNFI	PCFI
Reference	1–2	< 0.08	> 0.9	> 0.9	> 0.9	> 0.9	> 0.5	> 0.5	> 0.5
Initial model	2.302	0.043	0.887	0.872	0.924	0.932	0.884	0.784	0.824
Final model	1.778	0.033	0.915	0.901	0.954	0.961	0.859	0.786	0.825

### The effect relationship among a range of factors

The influence path analysis of each factor on acute exacerbation readmission of elderly patients with COPD within 30 days is shown in Fig. 2 and Table 4. Season, smoking, age, disease status, chronic disease, and overall health status had a positive relationship with 30-day acute exacerbation, whereas family rehabilitation had a negative effect on 30-day acute exacerbation in elderly patients with COPD. In addition, smoking indirectly impacted 30-day acute exacerbation through disease status, whereas disease status and chronic disease influenced 30-day acute exacerbation indirectly through a patient’s overall health status.

**Table 4:** Analysis of the influencing pathways of each factor on acute exacerbation readmission within 30 days

Variable	Acute exacerbation readmission within 30 days		
	Direct effect	Indirect effect	Total effect
Seasonal factors	0.089	–	0.089
Smoking status	0.084	0.010	0.094
Family rehabilitation	− 0.189	–	− 0.189
Age factors	0.378	–	0.378
Disease status	0.090	0.018	0.108
Chronic disease	0.600	0.018	0.618
Overall status	0.090	–	0.090

## Discussion

In this study, the prevalence of acute exacerbation readmission within 30 days of elderly patients with COPD was explored, and its influencing factors were analysed using a structural equation model. The main findings were that in this predefined population, the readmission rate of acute exacerbation within 30 days was 21.52%; the initial model based on literature and professional knowledge failed to meet the adaptation standard, whereas after in-depth consideration of the various parameters, the modified model met the standard required; season, smoking, age, disease status, chronic disease and overall health status were factors that influenced readmission, and family rehabilitation had a negative effect on readmission within 30 days. The novelty of the present study is that compared with conventional statistical methods, the structural equation can more effectively identify the factors related to the event. Furthermore, this method can describe each factor’s direct and indirect effects on the event, which is helpful for clinical decision-making and early intervention.

Airflow restriction in patients with COPD is often progressive due to the increased chronic inflammatory response of the airways and lungs to toxic particles or gases [1]. Studies have shown that acute exacerbations repeatedly occur in the development of COPD and may lead to readmission due to acute exacerbations in a short period after discharge [13]. There are few studies on acute exacerbation readmission of patients with COPD in China. Chen Hu et al. [14] showed that the 31-day readmission rate of patients with COPD in five general hospitals in Beijing was 2.67–6.3%. A study in Northeast China showed that the readmission rate of patients older than 18 years was 4.5% [15]. The results of this study showed that the rate of acute exacerbation readmission within 30 days was 21.5%, which was different from the results of other studies and may be related to the older age of the subjects in this study. With age, the immune system is weakened; the cough reflex is weakened, and with the coexistence of multiple diseases and malnutrition, these factors make the elderly a high-risk group for infection, and bacterial and viral infections are considered to be the most common causes of acute exacerbation in patients with COPD [16]. Studies outside of China have reported a 30-day readmission rate of patients with COPD of 6.70–7.54% and a readmission rate of elderly patients as high as 18.4% [3–5].

This study showed that factors relating to age and season, family rehabilitation and overall health status directly affected acute exacerbation readmission of elderly patients with COPD within 30 days. The effect values were 0.378, 0.089, -0.189 and 0.090, respectively, in which age factors were the main variables affecting readmission. Studies have shown that changes in the physiological aging of lung parenchyma and airways are similar to the changes in lung structure that are caused by the development of COPD [17], and the decline of lung function will cause a gradual deterioration of a patient’s condition, leading to frequent hospitalisation. Acute exacerbation of COPD is closely related to seasonal changes. Patients with low temperatures have obvious immune dysfunctions, and cold air stimulation can easily lead to chronic airway inflammation aggravation [18]. In addition, patients with COPD are primarily elderly whose airway responsiveness is not only increased but also more sensitive to air temperature changes, which easily causes airway spasms [19]. This study found that seasonal factors influenced the rate of acute exacerbation readmission. The colder the weather, the higher the rate of acute exacerbation readmission within 30 days of discharge, similar to the results that Fang et al. reported [20]. In this study, regular medication, rehabilitation exercise and home oxygen therapy were taken as indicators reflecting family rehabilitation, and the results showed that family rehabilitation had a direct negative effect on readmission. Home rehabilitation can improve a patient’s pulmonary ventilation function and effectively prevent the progression of the disease, thus reducing the rate of acute exacerbation readmission [21, 22]. Therefore, medical staff should strengthen their awareness and knowledge of patients’ medication, oxygen use and rehabilitation and optimise medication, oxygen use and exercise compliance. In this study, depression status, nutritional status and CAT, mMRC and DLA scores were used as indicators to reflect the overall health of the patient, and overall poor health had a direct positive effect on acute exacerbation readmission of elderly patients with COPD within 30 days. Relevant studies have shown that disease severity and the nutritional condition and emotional state of the patient affect the disease status, with poor values leading to frequent hospitalisation, which is similar to the results of this study [23]. Therefore, medical staff should pay attention to a patient’s overall health status as well as disease status to reduce the risk of readmission.

The results of this study showed that smoking not only had a direct influence on acute exacerbation of readmission within 30 days of elderly patients with COPD but also had an indirect effect through disease status, with a direct effect value of 0.084 and an indirect effect value of 0.010. Smoking was significantly associated with acute exacerbation readmission, and smokers had a higher risk of readmission than those who had quit smoking or never smoked. In addition, smoking is one of the critical risk factors causing a decline in lung function. After the oxidant in cigarette smoke enters the body, it stimulates the alveolar macrophages to produce excessive reactive oxygen species, thus causing the aggregation of inflammatory cells in the lungs and promoting an inflammatory response. This inflammatory response is particularly severe for small and terminal airways and can lead to lung tissue remodelling and ultimately to impaired lung function, which increases the risk of readmission [24]. Disease status is not only a direct factor influencing acute exacerbation readmission of elderly patients with COPD within 30 days but also an indirect factor through the patient’s overall health status, with a direct effect value of 0.090 and an indirect effect value of 0.018. Patients with COPD generally do poorly when it comes to daily living activities, and their nutritional condition and emotional state can be poor; these outcomes are due to increased energy consumption, electrolyte disorder, digestive disorder and the influence of drugs [25, 26]. Wang et al. [27] showed that the disease status of COPD affected the overall health status of patients, and the overall health status of patients in the acute stage were generally poor. Chronic disease is not only a direct factor affecting acute exacerbation readmission in elderly patients with COPD within 30 days but also an indirect factor affecting patients’ overall health status, with a direct effect value of 0.600 and an indirect effect value of 0.018. As a specific group, the elderly population is often affected by chronic diseases. According to the disease database released by the National Health Commission, the common chronic diseases in the elderly are hypertension, diabetes, CHD and hypoproteinaemia. Studies have shown that patients with chronic diseases have an increased risk of readmission [28]. Elderly patients with chronic diseases often need long-term medication and repeated hospitalisations due to prolonged treatments, resulting in weakness, poor nutritional statuses, decreased daily living ability, depression, pessimism and generally poor overall health statuses [29]. Recently, more attention has been paid to the factors directly influencing acute exacerbation readmission of patients with COPD within 30 days, but the interaction between independent variables has not been considered.

Other exacerbation models were proposed for patients with COPD in previous studies [30]. Compared with those models, our study has several distinct differences. First, each model predicts different time points of outcome events. Recently, Wu et al. [31] developed a novel COPD-readmission score (CORE) which included five predictors (eosinophil count, lung function, triple inhaler therapy, previous hospitalisation and neuromuscular disease) to predict one-year readmission after analysing 625 patients. The COPD-2-HOME score was designed for the prediction of a 90-day COPD-related hospital presentation [32]. Second, variables included in these studies were primarily from clinical scenarios such as laboratory and lung function tests, while the structural equation used in this study focused on epidemiological risk factors. The COPD-2-HOME score consists of five independent predictors: CAT score, hyperinflation, obstruction, prior admission and eosinophilia [32]. Third, the statistical methods used differ from study to study. The CORE score was developed by multivariable logistic regression, while Chen et al. [33] used an extreme gradient boosting (XGBoost) model to predict an acute exacerbation of COPD within one year. Our study constructed a structural equation model to predict 30-day COPD-related readmission that can also reflect the interaction among multiple variables.

There are some limitations to this study. Although our structural equation model contained seven latent variables, it only accounted for 61.11% of the total variation, indicating that there might be other factors associated with short-term readmission that were not identified in this study. Therefore, subsequent studies should be performed using systematic screening and comprehensive analyses of the influencing factors in COPD readmission. In addition, only thirteen hospitals in Ningxia were included in this study due to limited time, workforce and funds, leading to a relatively small sample size. Data from patients in other provinces and cities should be analysed to validate the generalisability of our results.

## Conclusion

In conclusion, the acute exacerbation readmission rate of elderly patients with COPD within 30 days was relatively high when compared with results from previous studies. Season, smoking, age, disease status, chronic disease and overall health status were factors influencing readmission within 30 days, whereas family rehabilitation had a reducing effect on readmission within 30 days. When taking measures to target these factors that directly influence readmission, attention should also be paid to the interaction among them.

## Data Availability

All data generated or analyzed during this study are included in this published article.
